# A T2* MRI prospective survey on heart iron in thalassemia major patients treated with sequential deferiprone-desferrioxamine versus deferipron and desferrioxamine in monotherapy

**DOI:** 10.1186/1532-429X-13-S1-P304

**Published:** 2011-02-02

**Authors:** Alessia Pepe, Antonella Meloni, Giuseppe Rossi, Maria Chiara Dell'Amico, Luciano Prossomariti, Domenico G D'Ascola, Aldo Filosa, Pasquale Pepe, Vincenzo Positano, Claudio Ascioti, Marcello Capra, Massimo Lombardi

**Affiliations:** 1 “G. Monasterio” Foundation and Institute of Clinical Physiology, CNR, Pisa, Italy; 2 A.O.R.N. Cardarelli, Napoli, Italy; 3 A.O. "Bianchi-Melacrino-Morelli", Reggio Calabria, Italy; 4 P.O. “Giovanni Paolo II”, Lamezia Terme, Italy; 5 P.O. G. Di Cristina, ARNAS Civico, Palermo, Italy

## Purpose

Most deaths in thalassemia major (TM) result from cardiac complications due to iron overload. No data are available in literature about possible different changes in cardiac iron in TM patients treated with sequential deferipron-deferoxamine (DFP-DFO) versus deferipron (DFP) and deferoxamine (DFO) in monotherapy. Magnetic Resonance (MR) is the unique non invasive suitable technique to evaluate quantitatively this issue. Our aim was to prospectively assess in the clinical practice the efficacy of the DFP-DFO versus DFP and DFO in monotherapy in a cohort of TM patients by quantitative MR.

## Methods

Among the first 739 TM patients enrolled in the MIOT (Myocardial Iron Overload in Thalassemia) network, 253 patients performed a MR follow up study at 18 ± 3 months according to the protocol. We evaluated prospectively the 25 patients treated with DFP-DFO versus the 30 patients treated with DFP and the 66 patients treated with DFO between the 2 MR scans. Myocardial iron concentrations were measured by T2* multislice multiecho technique.

## Results

**Figure 1:**
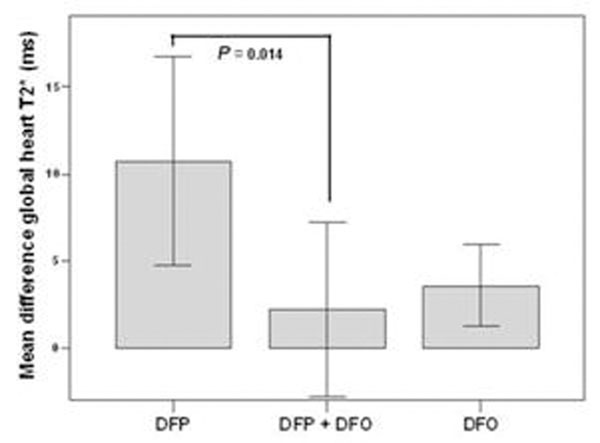


Excellent/good levels of compliance were similar in the 3 groups (DFP-DFO 96% versus DFP 97% versus DFO 92%; *P*=0.67). Among the patients with no significant myocardial iron overload (MIO) at baseline (global heart T2* ≥ 20 ms), there were no significant differences between groups to maintain the patients without MIO (DFP-DFO 95% versus DFP 100% versus DFO 100%; *P*=0.23). Among the patients with MIO at baseline (global heart T2* < 20 ms), only DFP and DFO showed a significant improvement in the global heart T2* value (*P*=0.001 and *P*=0.003, respectively) and in the number of segment with a normal T2* value (*P*=0.031 and *P*=0.0001, respectively). The improvement in the global heart T2* was significantly different among groups (mean difference global heart T2* DFP-DFO 2.2 ± 4.1 ms, DFP 10.7 ± 7.2, DFO 3.6 ± 5.4; *P*=0.007). The improvement in the global heart T2* was significantly lower in the DFP-DFO versus DFP group (*P*=0.014), but it was not significantly different in the DFP-DFO versus the DFO group (P=0.63) (see figure 1).

## Conclusions

Prospectively in a clinical setting over 15 months we did not find significant differences on cardiac iron in TM patients treated with sequential DFP-DFO versus the TM patients treated with DFO. Conversely, DFP monotherapy was significantly more effective than DFP-DFO in improving myocardial siderosis.

